# The Evaluation and Prediction of Flame Retardancy of Asphalt Mixture Based on PCA-RBF Neural Network Model

**DOI:** 10.3390/ma17133298

**Published:** 2024-07-04

**Authors:** Peng Yin, Haowu Wang, Yangwei Tan

**Affiliations:** 1 School of Infrastructure Engineering, Dalian University of Technology, No. 2, Linggong Road, Ganjingzi District, Dalian 116024, China; yp2021@mail.dlut.edu.cn; 2 School of Civil Engineering, Chongqing Jiaotong University, 66 Xuefu Ave., Nan’an District, Chongqing 400074, China; 3 Department of Civil and Airport Engineering, Nanjing University of Aeronautics and Astronautics, Nanjing 211106, China; tanyw07@nuaa.edu.cn

**Keywords:** flame retardant, pavement performance, flame retardancy, RBF neural network, finite element model

## Abstract

Warm mix flame retardant asphalt mixture can reduce the energy dissipation and harmful gas emissions during asphalt pavement construction, as well as mitigate the adverse effects of road fires. For this, this paper studies the design and performance of a mixture modified with a combination of warm mix agent and flame retardant, and the pavement performance and flame retardancy of the modified mixture are evaluated. Additionally, a flame retardancy prediction model based on the radial basis function (RBF) neural network model is established. On this basis, the principal components analysis (PCA) model is used to analyze the most significant evaluation indicators affecting flame retardancy, and finally, a three-dimensional finite element model is developed to analyze the effects of loading on the pavement structure. The results show that compared to virgin asphalt mixture, the modified mixture shows a reduction in mixing and compaction temperatures by approximately 12 °C. The high-temperature performance of the mixture is improved, while the low-temperature performance and moisture stability slightly decrease, but its flame retardancy is significantly enhanced. The RBF neural network model revealed that the established flame retardancy prediction model has a high accuracy, allowing for precise evaluation of the flame retardancy. Finally, the PCA model identified that the combustion time has a significant effect on the flame retardancy of the asphalt mixture, and the finite element model revealed that the displacements of the warm mix fire retardant asphalt mixture were lower than virgin asphalt mixture in all directions under the loading.

## 1. Introduction

With the continuous development of the economy and construction projects, the number of tunnels in use is steadily increasing [1,2]. However, as tunnel traffic increases, the incidence of related fire accidents is also rising. Asphalt combustion releases a large amount of heat, which is difficult to dissipate, and the asphalt fumes in tunnels reduce visibility [3,4]. The occurrence of fire accidents not only poses a threat to people’s safety but also causes significant damage to roads. Due to the unique external conditions, the construction environment during the construction process, and the special conditions of vehicle use in tunnels, the quality standards for tunnel construction have been correspondingly increased. Flame retardants, which can be incorporated into flammable materials to prevent fires and suppress the extent of fire spread, can effectively enhance the thermal stability and flame retardancy of asphalt when mixed with it [5,6,7]. This reduces the likelihood of asphalt combustion and increases the safety of asphalt pavements in tunnels. Therefore, flame retardants have become an important means of advancing the development of flame retardant asphalt pavements [8,9].

Flame retardant asphalt exhibits higher thermal stability and aging resistance, maintaining good performance in high-temperature environments. Traditional asphalt tends to age, soften, or even melt under high temperatures and in the presence of fire, leading to pavement damage and reduced lifespan. Flame retardant asphalt pavements can effectively resist high temperatures and fire erosion, extending the service life of roads, reducing the frequency of road maintenance and reconstruction, and thereby lowering maintenance costs [10,11,12]. Specifically, Liu [13] combined an environmentally friendly flame retardant and polyurethane to modify asphalt, and found that the high- and low-temperature performance and flame retardancy of asphalt were significantly improved, and there was a certain synergistic effect between different flame retardant components. Zhao [14] used six kinds of flame retardant to prepare flame retardant asphalt, and discovered that the incorporation of flame retardant can effectively improve the thermal stability and flame retardancy of asphalt. Furthermore, the addition of styrene–butadiene rubber could further enhance the paving performance of the asphalt. Wang [15] prepared an inorganic flame retardant to develop flame retardant asphalt to alleviate the toxicity of organic flame retardants, and found that the flame retardant asphalt exhibited significantly improved combustion characteristics and thermal stability compared to base asphalt. Wu [16] investigated the effect of mixed flame retardants on the flammability and mechanical properties of asphalt, and found that within a certain dosage range, the mixed flame retardants could improve the asphalt flame retardancy performance by depleting the heat released from the asphalt. Tan [17] explored the synergistic flame retardant system of nanomaterials and conventional flame retardants on the flame retardancy of asphalt. It was found that the synergistic effect of the two could further enhance the flame retardancy of asphalt, and the carbon layer formed by the decomposition of flame retardants within the asphalt could effectively protect it. These research results indicate that significant progress has been made in the application of flame retardants in asphalt pavements, establishing flame retardants as an effective means to improve the flame retardancy of asphalt.

However, traditional asphalt often requires high temperatures during construction and paving, which poses health and safety risks to construction workers. In addition, the fumes and harmful gases produced during high-temperature operations can also harm the respiratory system of workers. In particular, flame retardants are generally powder materials that require high temperatures during the production of flame retardant asphalt to achieve effective blending with asphalt [18,19]. Consequently, researchers have increasingly favored warm mix flame retardant asphalt technology. Li [20] analyzed the synergistic effects of warm mix agent and flame retardant on the service performance of asphalt. It was found that flame retardant effectively enhanced the flame retardancy of asphalt, while the addition of warm mix agent significantly reduced the high-temperature viscosity of asphalt. Furthermore, the synergistic effects of both could further improve the flame retardancy of asphalt. Jiang [21] investigated the viscosity reducing effect of warm mix agent on flame retardant asphalt and found that warm mix agent could effectively decrease the high-temperature viscosity of asphalt and improve its flowability. Li [22] investigated the effects of composite flame retardant and warm mix agent on the flame retardancy of asphalt, and found that the incorporation of warm mix agent does not significantly affect the flame retardancy of asphalt, and the warm mix agent can also shorten the ignition time of asphalt and reduce the heat released. Xia [23] revealed the synergistic inhibition mechanism of warm mix agent and flame retardant on the combustion behavior of asphalt, and found that the incorporation of warm mix agent can facilitate the flame retardant in providing a better flame retardant effect. Based on the research results above, it can be observed that the synergistic effect of warm mix agent and flame retardant not only facilitates further enhancement of asphalt’s flame retardancy but also reduces the high-temperature viscosity of asphalt. Therefore, the mixing temperature of asphalt mixtures is reduced, and the heat release and greenhouse gas emission during asphalt pavement paving is lowered. However, it is noted that current research on warm mix flame retardant technology primarily focuses on asphalt, with relatively limited evaluation of flame retardancy in asphalt mixtures. To further promote the development of warm mix flame retardant asphalt, it is necessary to conduct further research on the flame retardancy of warm mix flame retardant asphalt mixture.

Warm mix flame retardant asphalt mixtures are primarily used in tunnel pavements, where their quality directly impacts the safety, durability, and social benefits of the roadway. Currently, the market offers a wide range of flame retardants with varying effectiveness and lack of specificity. This paper employs the response surface methodology to design the formulation of flame retardants rationally. Performance tests are conducted to determine the optimal dosage of warm mix agent and flame retardant. Subsequently, pavement performance tests are performed to verify the pavement performance of the warm mix flame retardant asphalt mixture. While ensuring the pavement performance, the flame retardant effect of the mixture is comprehensively evaluated. Subsequently, MATLAB R2021a software is used to establish an RBF neural network model to predict and evaluate the flame retardancy. Based on this model, a PCA model is employed to analyze the significance of the evaluation indicators. This approach aims to provide insights and references for the further promotion and application of warm mix flame retardant asphalt mixture.

## 2. Materials and Methods

### 2.1. Asphalt

The virgin asphalt (VA) is SBS-modified asphalt from Guangdong, China, and its technical properties are shown in Table 1.

### 2.2. Aggregate

The aggregate selected in this paper is limestone, and its properties are shown in Table 2.

### 2.3. Warm Mix Agent

The warm mix agent used in this study is Energy Champion 120 (EC) warm mix agent from Guangdong, China, which has relatively stable properties and is commonly used in the construction of warm mix asphalt pavement. Its performance indicators are shown in Table 3.

### 2.4. Flame Retardant

The flame retardant developed in this study is primarily composed of magnesium hydroxide, diatomite, and aluminum hydroxide. This is because both hydroxides decompose at high temperatures and release water molecules, absorbing a large amount of heat, lowering the asphalt’s temperature, slowing down the combustion process, and forming a protective layer that prevents flame spread. As a porous material, diatomite can absorb and disperse magnesium hydroxide and aluminum hydroxide, enhancing the flame retardant effect. Additionally, its high-temperature stability can provide protection during a fire. Several conventional performance indicators and the limiting oxygen index were used as evaluation indicators. The response surface methodology was employed to establish a response surface model for computational analysis. The final formulation of the flame retardant was determined to be 21.511% of magnesium hydroxide, 4.923% of diatomaceous earth, and 4.105% of aluminum hydroxide. The prepared flame retardant is named FR. The basic properties of several materials are shown in Table 4. The specific preparation process of flame retardant is as follows. Take approximately 240 g of VA and keep it in the oven at 140 °C for about 2 h, then place it in an oil bath at 175 °C. Use a high-speed shearing machine to shear the asphalt. Gradually adjust the shearing machine’s speed from 0 to 5000 rpm. Sequentially add the pre-prepared flame retardant materials into the shearing machine. Continue the shearing process for 30 min, ultimately yielding the prepared flame retardant asphalt.

### 2.5. Gradation Design

**Figure 1:**
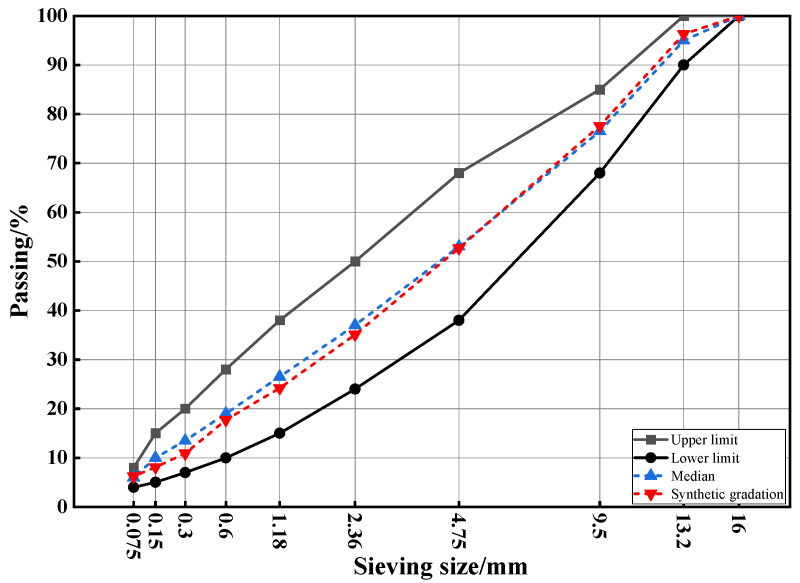
The results of gradation design.

In this study, the AC-13 gradation is used, with limestone as both the aggregate and mineral powder. The mix design is conducted according to the JTG F40-2004 standard [24]. The gradation design results are shown in Figure 1. The optimal asphalt–aggregate ratio for the warm mix flame retardant asphalt mixture is calculated to be 5.1%, while the ratio for the virgin asphalt mixture is 4.7%.

### 2.6. Preparation of Asphalt Mixture

To investigate the change trend in the flame retardancy of the mixture before and after modification, this study uses a wet modification process to prepare the warm mix flame retardant asphalt mixture. First, the warm mix flame retardant asphalt is prepared according to the preparation process described earlier. Then, based on relevant research experience [25], the aggregate is heated to a dry state in an oven at 100 °C. The aggregate is then placed in a mixer, and a certain amount of asphalt is added to the mixer. The mixture of asphalt and aggregate is thoroughly mixed at the mixing temperature calculated from the viscosity–temperature curve. After mixing, the mixture is placed in different molds to prepare the desired asphalt mixture specimens.

### 2.7. Methods

#### 2.7.1. Conventional Performance Test

To prepare warm mix fire retardant asphalt mixtures with excellent performance, FR and EC are used in this study to prepare warm mix flame retardant asphalt, and to investigate the optimal dosage of FR and EC, the physical properties of the asphalt before and after modification are evaluated by using penetration, ductility, softening point, and rotational viscosity (RV) tests in this study [26,27]. Based on JTG E20-2011 [28], the test temperature for the penetration test is 25 °C, for the ductility test it is 5 °C, and for the RV test it is 135 °C.

#### 2.7.2. Limiting Oxygen Index (LOI) Test

To evaluate the effect of FR and EC dosage on the flame retardancy of asphalt, the LOI test is used to evaluate the flame retardancy of several asphalts [29,30]. The initial oxygen concentration is 20%, the test gases are oxygen and nitrogen, and the gas flow rate is 40 mm/s ± 10 mm/s. 

#### 2.7.3. Thermogravimetry (TG) Test

To evaluate the thermal stability of asphalt at high temperatures, this study conducted the TG test on several asphalts. The test temperature ranged from 0 to 800 °C with a heating rate of 10 °C/min, using argon gas as the protective gas.

#### 2.7.4. Differential Scanning Calorimetry (DSC) Test

To further characterize the temperature stability of several asphalts at low temperatures and assess the influence of modification on the thermal properties of asphalt, this study conducted the DSC test on several asphalts. The test temperature ranged from −80 to 200 °C with a heating rate of 10 °C/min, using argon gas as the protective gas.

#### 2.7.5. Gas Chromatography–Mass Spectrometry (GC-MS) Test

Asphalt releases a large amount of asphalt smoke during combustion, which contains many volatile organic compounds (VOCs). To detect the inhibitory effect of warm mix flame retardant modification on VOC release, VOCs were collected during the combustion process of two asphalts. The VOC samples were then analyzed using the GC-MS method. After the GC-MS test, qualitative analysis was performed by matching the characteristic ions of target compounds with those in the NIST mass spectroscopy library. Calibration was conducted using the least squares method, with the correlation of the analysis results with the internal standard being greater than 0.99.

#### 2.7.6. Pavement Performance Test

To characterize the impact of FR and EC on the pavement performance of asphalt mixture, the high-temperature performance, low-temperature performance, and moisture stability of the mixture are evaluated by the rutting test, beam bending test, and freeze–thaw splitting test, respectively [31]. According to JTG E20-2011 [28], the temperature of the rutting test is 60 °C, and the rolling speed is 42 ± 1 times/min, and the high-temperature performance is evaluated by the deformation of rutting and dynamic stability. The temperature of the beam bending test is −10 °C, and the low temperature performance is evaluated by bending and tensile strength and the maximum failure strain. For the freeze–thaw splitting test, the specimens are divided into two groups. One group of specimens is first saturated with water for 15 min, then frozen at −18 °C for 16 h, followed by being placed in a water bath at 60 °C for 24 h. After the water bath, the specimens are transferred to a water bath at 25 °C and maintained for 2 h before testing. The other group of specimens serves as the control group. After the test, moisture stability is evaluated based on the splitting strength and the freeze–thaw splitting strength ratio (TSR).

#### 2.7.7. Combustion Test

Based on the relevant research experience, the combustion behavior and flame retardancy of asphalt mixtures are mainly evaluated by combustion test [32,33]. The specimens used in the combustion test are Marshall specimens, the specimens are placed in the iron plate, each specimen is poured into 50 mL of gasoline, then ignited, and the test results are recorded after combustion. After the test, the flame retardancy of asphalt mixtures is evaluated based on the combustion time, mass loss rate, and the change in stability.

## 3. Results

### 3.1. Design of Warm Mix Flame Retardant Asphalt

**Figure 2:**
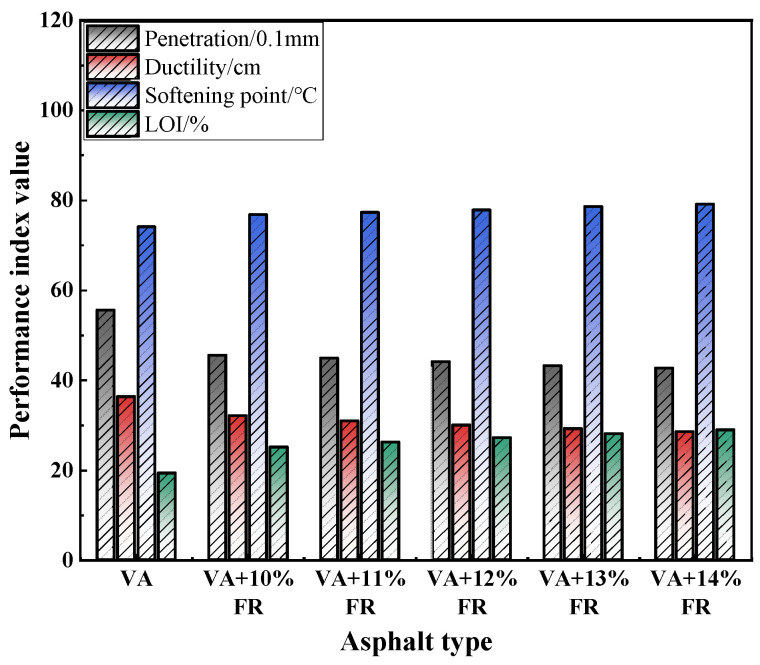
Performance index of flame retardant asphalt.

To determine the appropriate dosage of FR, five different dosages (10%, 11%, 12%, 13%, and 14%) were selected and incorporated into VA. Following the previously described preparation process, five groups of flame retardant asphalt with different dosages were prepared. These groups were then subjected to a conventional performance test. The test results are shown in Figure 2.

From Figure 2, it can be observed that as the dosage of the flame retardant increases, the penetration and ductility values show a decreasing trend, while the softening point and LOI show an increasing trend. Compared to VA, regardless of the flame retardant dosage, both the penetration and ductility values decrease significantly, with the minimum penetration value approaching the specification limit. When the flame retardant dosage is 10% or 11%, the reduction in ductility is relatively low. However, the LOI values are too low and still fall within the range of flammable materials, failing to meet usage requirements. While the LOI values for the flame retardant asphalt with dosages of 12%, 13%, and 14% meet the usage requirements, their ductility and penetration values are relatively lower compared to the former. Therefore, comprehensive analysis suggests that the optimal dosage of FR is 12%.

**Figure 3:**
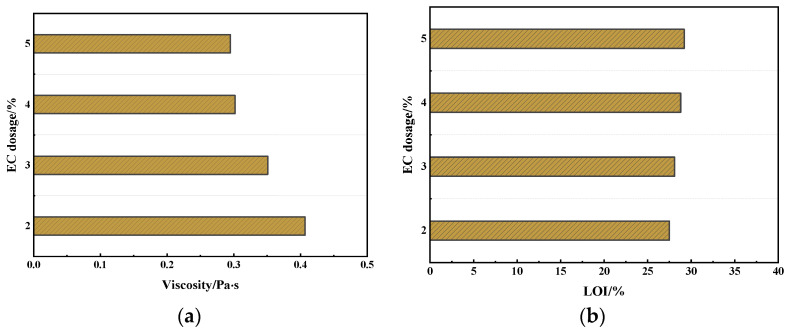
Test results of viscosity and LOI: (**a**) viscosity; (**b**) LOI.

The warm mix modification of asphalt aims to reduce viscosity, thereby lowering the mixing and compaction temperatures of the mixture. The LOI characterizes the flame retardancy of the material. Thus, this study determines the optimal dosage of the EC based on the comparative analysis of relevant test indicators. The test results are shown in Figure 3.

From Figure 3, it is evident that as the dosage of EC increases, the viscosity of the asphalt decreases, indicating that the addition of EC effectively reduces the viscosity of the asphalt. Additionally, the LOI shows an increasing trend, suggesting that EC synergizes with FR, improving the flame retardancy of the asphalt. However, the test results indicate that beyond a certain dosage, the improvements in both flame retardancy and viscosity reduction effect become marginal. Therefore, there is an optimal dosage for EC. According to the test results, when the dosage exceeds 4%, the modification effects of EC significantly diminish. Consequently, the optimal dosage of EC is determined to be 4%.

**Figure 4:**
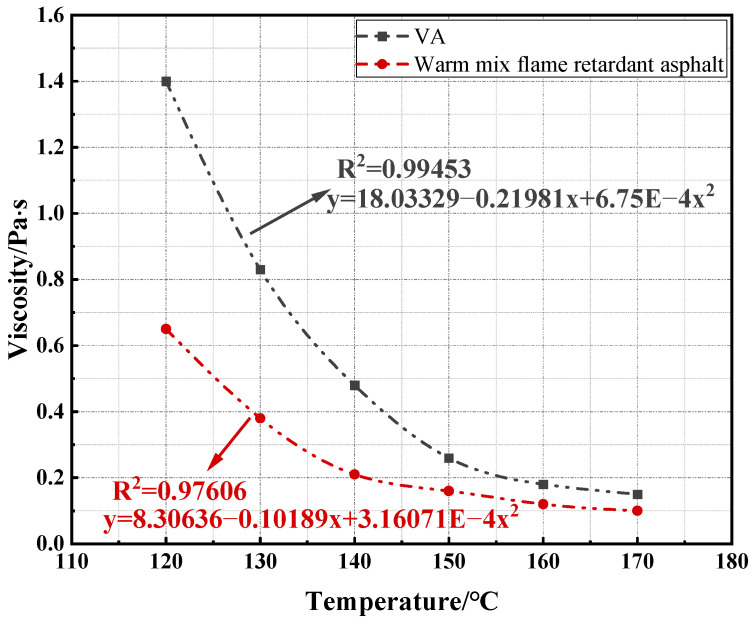
Viscosity–temperature curve of asphalt.

To determine the mixing and compaction temperature of the mixture, the viscosity–temperature curve method is typically used. This involves identifying the temperatures on the viscosity–temperature curve that correspond to viscosity of 0.17 ± 0.02 Pa·s and 0.28 ± 0.03 Pa·s [34], which are used as the mixing and compaction temperatures, respectively. Therefore, this study calculates and analyzes the viscosity-reducing effect of the warm mix additive using viscosity–temperature curves. The results of the viscosity–temperature curves are shown in Figure 4.

From Figure 4, it can be observed that as the temperature increases, the viscosity decreases. Additionally, under the same temperature conditions, the viscosity of warm mix flame retardant asphalt is significantly lower than VA. This indicates that the use of the warm mix agent effectively improves the high-temperature viscosity of the asphalt, thereby reducing the construction temperature of the asphalt pavement. Then, a fitting analysis was performed on the viscosity–temperature curve of VA, and the equation obtained is y = 18.03329 − 0.21981x + 0.000675x^2^. When the y values are 0.15, 0.19, 0.25, and 0.31, the corresponding x values are 167, 154, 150, and 147, respectively. Therefore, the mixing temperature range for VA is 154~167 °C, and the compaction temperature range is 147~150 °C. Fitting the viscosity–temperature curve for the warm mix flame retardant asphalt, the equation obtained is y = 8.30636 − 0.10189x + 0.000316071x^2^. When the y values are 0.15, 0.19, 0.25, and 0.31, the corresponding x values are 148, 144, 139, and 135, respectively. Therefore, the mixing temperature range for warm mix flame retardant asphalt is 144~148 °C, and the compaction temperature range is 135~139 °C. From this, it can be seen that the mixing and compaction temperatures of the warm mix flame retardant asphalt mixture are reduced by approximately 12 °C. This indicates that the addition of a warm mix agent can lower the mixing and compaction temperatures of the asphalt mixture, achieving the warm mix effect.

### 3.2. Thermal Stability Analysis of Warm Mix Flame Retardant Asphalt

**Figure 5:**
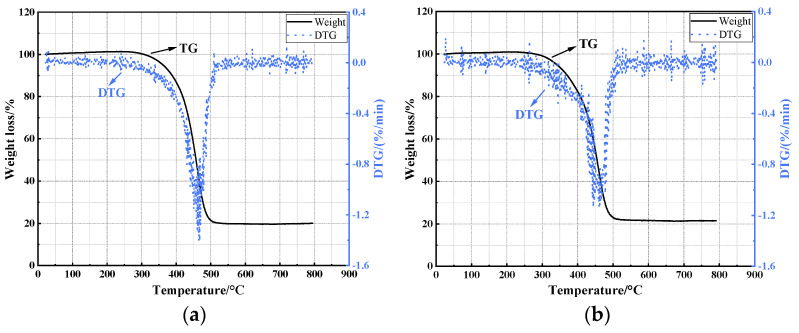
The results of TG test: (**a**) VA; (**b**) warm mix flame retardant asphalt.

To further evaluate the thermal stability of warm mix flame retardant asphalt, the TG test was conducted on several asphalts, which is illustrated in Figure 5.

From Figure 5, it can be observed from the DTG curves of the two asphalts that the weight loss rate of asphalt has a clear step change in the early stage, and there is basically no weight loss of asphalt from 0 °C to 300 °C. Asphalt can maintain its performance stability in the low-temperature region, which also microscopically proves that the quality of asphalt experiences virtually no loss during heating and mixing processes, making it suitable for use in actual production. Starting at around 300 °C, significant changes in the endothermic and exothermic peaks occur. This indicates that the light components of the asphalt begin to volatilize, leading to a corresponding decrease in asphalt mass. This temperature can be considered the flash point, as it is consistent with the temperature at which mass loss occurs. The TG analysis results of the asphalt meet the flash point index standard, demonstrating a correlation between the macroscopic and microscopic properties of the asphalt. The temperature range and loss rate of asphalt mass loss are influenced by the types of asphalt. From the TG and DTG curves of the two asphalts, it is evident that in the temperature range of 350 °C to 500 °C, the mass loss rate of VA is significantly higher than warm mix flame retardant asphalt. This aligns with the results of the flame retardants improving the high-temperature performance of the asphalt. Additionally, according to the DTG curve, the maximum weight loss rate is positively correlated with the light components of the asphalt. The maximum weight loss rate increases with the softening point. If the softening point is higher, the melting temperature also increases, resulting in a higher temperature for the volatilization of the light components.

**Figure 6:**
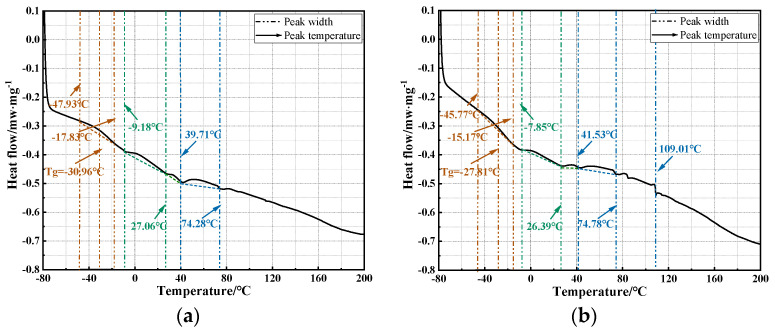
The results of DSC test: (**a**) VA; (**b**) warm mix flame retardant asphalt.

Additionally, to further analyze the impact of the warm mix flame retardant modification on the thermal stability of asphalt, the DSC test was conducted on the two asphalts. The results are shown in Figure 6.

From Figure 6, compared to VA, the glass transition temperature (T_g_) of warm mix flame retardant asphalt is significantly lower. T_g_ represents the temperature at which asphalt transitions from a glassy state to a viscoelastic state. When the temperature is below T_g_, the asphalt is in a glassy state; when the temperature is above T_g_, the asphalt is in a viscoelastic state. Therefore, T_g_ can also characterize the low-temperature performance of asphalt to a certain degree. It can be observed that the use of warm mix agent and flame retardant significantly lowers the low-temperature stability of the asphalt, making it easier for the asphalt to transition from a glassy state to a viscoelastic state. This is because flame retardant is essentially composed of powder materials. When mixed with asphalt, it increases the asphalt’s viscosity and absorbs some of the light components within the asphalt, thereby increasing its brittleness at low temperature. Consequently, the low-temperature stability of the asphalt is adversely affected, which is consistent with the results of the conventional performance tests. Notably, it can be observed that compared to VA, the endothermic peak area of warm mix flame retardant asphalt is significantly increased. This value characterizes the low-temperature stability of the asphalt; the higher the value, the poorer the stability. This indicates that while the warm mix flame retardant modification improves the thermal stability of the asphalt, the properties of the modification materials adversely affect the low-temperature stability of the asphalt. This is consistent with the previous analysis.

**Figure 7:**
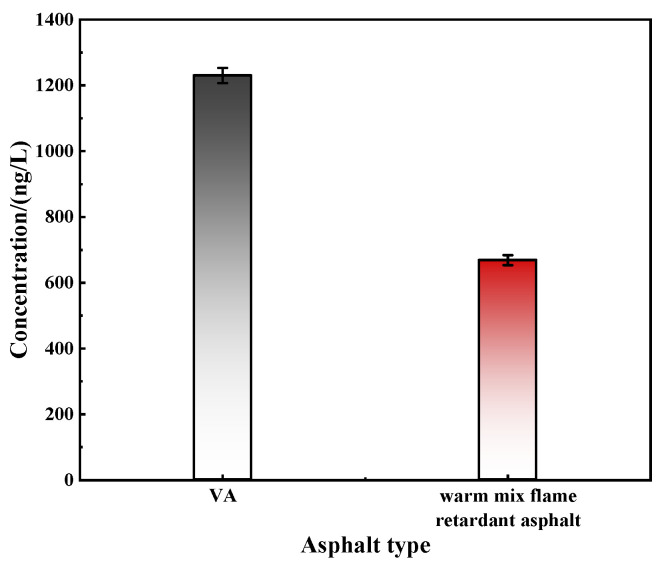
The VOC concentration of two asphalts.

Notably, although the TG and DSC tests have already verified the impact of warm mix agent and flame retardant on the thermal stability of asphalt, this study further characterizes the flame retardancy of warm mix flame retardant asphalt. Notably, although the effects of warm mix agent and flame retardant on the thermal stability of asphalt have been verified by TG and DSC tests, to further characterize the flame retardancy of warm mix flame retardant asphalt, this study combined the results of gas chromatography–mass spectrometry (GC-MS) to detect the volatile organic compound (VOC) components emitted from the two asphalts and described ten hazardous substances with high concentrations, which are shown in Figure 7 and Table 5.

From Figure 7 and Table 5, it can be observed that compared to VA, the VOC concentration in warm mix flame retardant asphalt is significantly decreased. This indicates that the warm mix agent and flame retardant used in this study can effectively reduce the emission of harmful gases from asphalt. This is because the warm mix agent lowers the required temperature for asphalt mixture mixing and compaction. The reduced temperature limits the volatilization process of asphalt at high temperatures. Additionally, the flame retardant primarily consists of hydroxides, which decompose at high temperatures and release water molecules, effectively curbing the volatilization of asphalt. Moreover, the diatomite in the flame retardant is a porous material that can absorb and disperse the hydroxides, thereby enhancing their flame retardant effect. Notably, Table 5 further elucidates the roles of the warm mix agent and flame retardant. Compared to VA, the overall VOC concentration in the warm mix flame retardant asphalt is significantly lower, and the concentrations of each harmful substance that constitutes the VOCs also show a clear downward trend. This further validates the flame retardancy of the warm mix flame retardant asphalt used in this study.

### 3.3. Evaluation of Pavement Performance of Mixture

**Figure 8:**
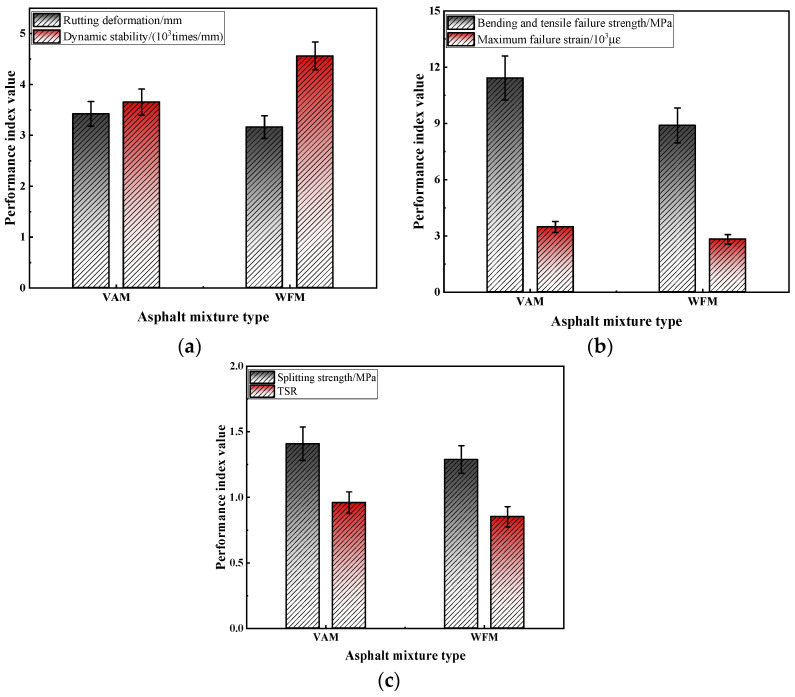
The pavement performance test results: (**a**) rutting test; (**b**) beam bending test; (**c**) freeze–thaw splitting test.

In this paper, the rutting test, beam bending test, and freeze–thaw splitting test are used to evaluate the pavement performance of warm mix flame retardant asphalt mixture (WFM) and virgin asphalt mixture (VAM) [35]; the test results are calculated and analyzed, as shown in Figure 8.

From Figure 8, it can be seen that after warm mix flame retardant modification of VAM, the high-temperature deformation resistance of the mixture is significantly improved. Compared to VAM, the dynamic stability of WFM increased by 24.9%, indicating that the warm mix flame retardant modification effectively enhances the high-temperature performance of the mixture. Secondly, compared to VAM, the bending and tensile strength and maximum failure strain of WFM show a decreasing trend at −10 °C. This indicates that the warm mix flame retardant modification weakens the low temperature crack resistance of VAM, making it more prone to cracking at lower temperatures. Although the low-temperature crack resistance decreases after the warm mix flame retardant modification of VAM, the test results still meet the requirements of JTG F40-2004 [24]. Therefore, the ratio of warm mix flame retardant asphalt obtained in this study is feasible from the perspective of low-temperature performance. Additionally, after the warm mix flame retardant modification of VAM, the moisture stability indicators of the mixture show a declining trend. This indicates that the warm mix flame retardant modification weakens the moisture stability of the mixture, making it more susceptible to water damage at lower temperatures. The analysis suggests that this may be because the warm mix flame retardant asphalt reduces the mixing temperature of the asphalt mixture, preventing the moisture in the asphalt from being fully expelled during the mixing and compaction process due to the lower temperatures. Notably, the warm mix agent used in this study is essentially a granular material. Granular warm mix agent has a smaller surface area and limited contact area with asphalt and aggregates. Their surface activity is not as effective as that of liquid warm mix agent, thereby affecting the moisture stability of the mixture. Additionally, granular warm mix agent may create tiny pores or channels within the mixture. These pores or channels can serve as pathways for water infiltration, leading to a decrease in the moisture stability of the mixture. Although the moisture stability decreases after the warm mix flame retardant modification of VAM, the test results still meet the requirements of JTG F40-2004 [24]. Therefore, the ratio of the warm mix flame retardant asphalt obtained in this study is feasible from the perspective of moisture stability.

### 3.4. Evaluation of Flame Retardant Effect

In this study, the flame retardant effect of the asphalt mixture is evaluated using a combustion test. This test simulates a roadway fire that could occur if vehicle fuel leaks onto the pavement. The flame retardancy of the asphalt mixture is assessed through the combustion test. The evaluation criteria include the combustion time, mass loss rate, and stability of the asphalt mixture after burning.

#### 3.4.1. Combustion Time

**Figure 9:**
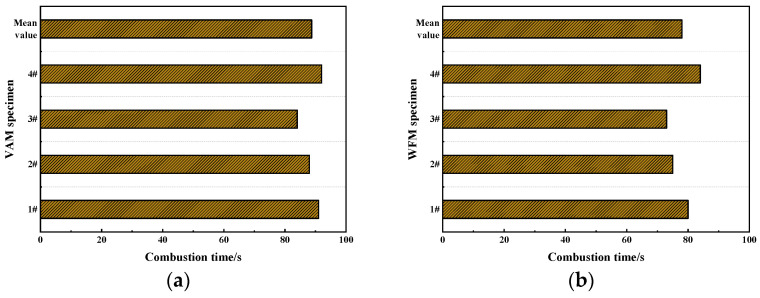
The combustion time of several asphalt mixtures: (**a**) VAM; (**b**) WFM.

The simulated combustion test was conducted on the VAM specimen as well as WFM specimen, and the test results are shown in Figure 9.

From Figure 9, it can be seen that the combustion time of WFM specimens is generally lower than VAM specimens. This is because the FR is uniformly dispersed in the asphalt. When the asphalt begins to burn, the FR is exposed to the combustion environment and acts to retard the flammable and combustible substances in the combustion environment. This indicates that the FR developed in this study can effectively retard the combustion of asphalt, a flammable material. Although the combustion times of some individual specimens of both mixtures are similar, this may be due to the primary combustion occurring on the gasoline on the surface of the asphalt mixture specimens, with the asphalt itself burning less. The addition of FR can only inhibit the combustion of the asphalt and has limited effectiveness in retarding the gasoline on the surface of the asphalt.

#### 3.4.2. Mass Loss Rate

**Figure 10:**
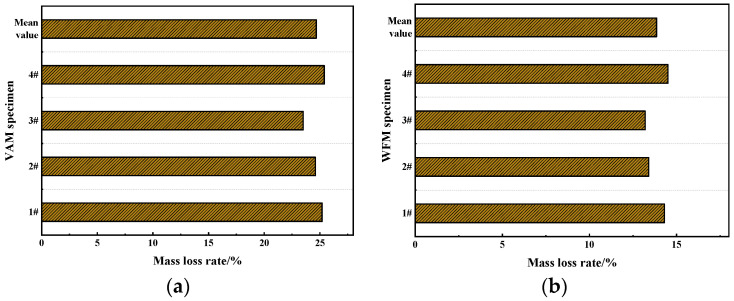
The mass loss rate of several asphalt mixtures: (**a**) VAM; (**b**) WFM.

To further investigate the flame retardancy of the two mixtures, the mass loss rate of the two mixtures after combustion was calculated, as shown in Figure 10.

From Figure 10, compared to VAM, the mass loss of the WFM specimens significantly decreased. The mass loss of WFM specimens was reduced by approximately 43.9%. This reduction is primarily because the flame retardant, encapsulated within the asphalt, is exposed to the high-temperature oxidation environment during combustion, thereby participating in the combustion process. This action mitigates the amount of asphalt involved in the combustion, resulting in reduced mass loss of the mixture after combustion. This indicates that the FR developed in this study effectively improves the flame retardancy of the asphalt mixture. During the combustion of asphalt, a large number of aromatic hydrocarbons such as anthracene, phenanthrene, and pyrene are produced, along with carcinogenic substances like benzopyrene, posing significant health hazards. This is especially critical in confined environments like tunnels, where the harmful gases generated by asphalt combustion cannot be promptly vented, causing severe physiological harm to personnel. The WFM effectively reduces mass loss after combustion, decreasing the decomposition of asphalt during the combustion process, and consequently lowering the emission of harmful gases.

#### 3.4.3. Stability

**Figure 11:**
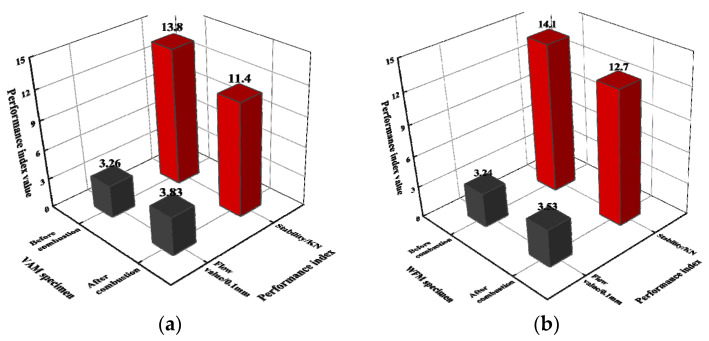
The stability of several asphalt mixtures: (**a**) VAM; (**b**) WFM.

To further evaluate the flame retardancy of the two mixtures, the stability of the two mixtures before and after combustion was tested and is shown in Figure 11.

Figure 11 shows that the residual stability of the VAM specimen is 82.61%, while the residual stability of the WFM specimen is 90.07%. This is because the stability of asphalt mixtures primarily comes from the interlocking effect of aggregates. During the combustion of the specimen, only the asphalt decomposes due to burning, reducing the binding effect of the asphalt and thereby lowering the stability of the mixture. In the WFM, the FR plays a role in inhibiting combustion during the asphalt combustion process, reducing the consumption of asphalt. As a result, the WFM retains relatively high stability during combustion, thus improving the flame retardancy of the asphalt mixture.

### 3.5. Prediction and Evaluation of Flame Retardancy

The previous combustion tests demonstrated that the incorporation of flame retardant and warm mix agent effectively enhanced the flame retardancy of the asphalt mixture, curbing its combustion behavior in fire-prone environments. However, although the combustion test can effectively characterize the role of flame retardant and warm mix agent, it cannot accurately predict the trend of changes in the flame retardancy of the asphalt mixture.

**Figure 12:**
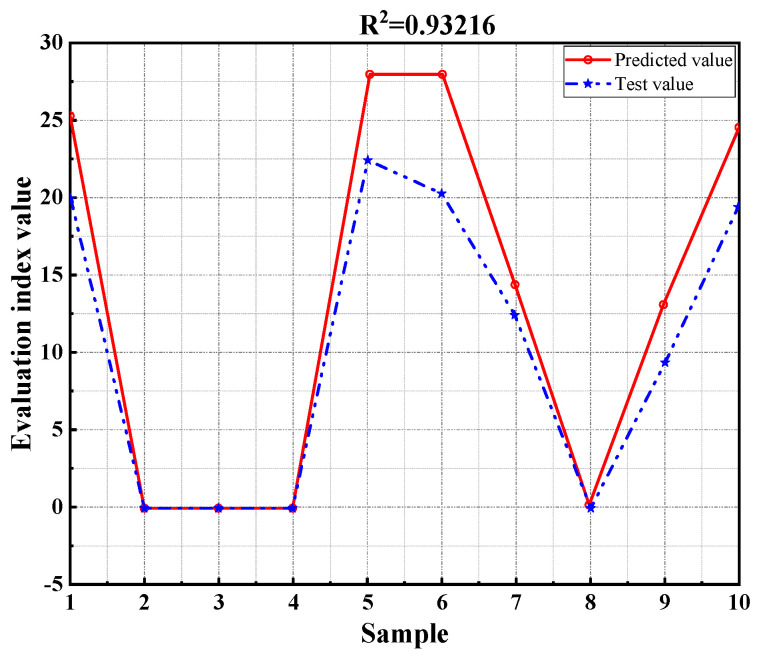
The prediction results of RBF neural network model.

In recent years, with the continuous development and application of various decision-making methods, researchers have begun integrating approaches like gray theory into road engineering, leading to interdisciplinary fusion and effectively solving numerous technical challenges. Against this backdrop, this study utilizes MATLAB software to develop a Radial Basis Function (RBF) neural network model [36,37], using it as the foundational algorithm for predicting flame retardant performance. It is worth noting that, to ensure the accuracy of the neural network model predictions, this study conducted ten independent calculations to reduce the impact of randomness on the results, and averaged the results from these calculations. This approach enabled this study to obtain more stable and reliable predictions, and further validate the stability and accuracy of the predictive model. The model selects combustion time, mass loss rate, and stability as evaluation indicators, incorporating mixture type as a parameter. The results of the model calculations are shown in Figure 12.

**Figure 13:**
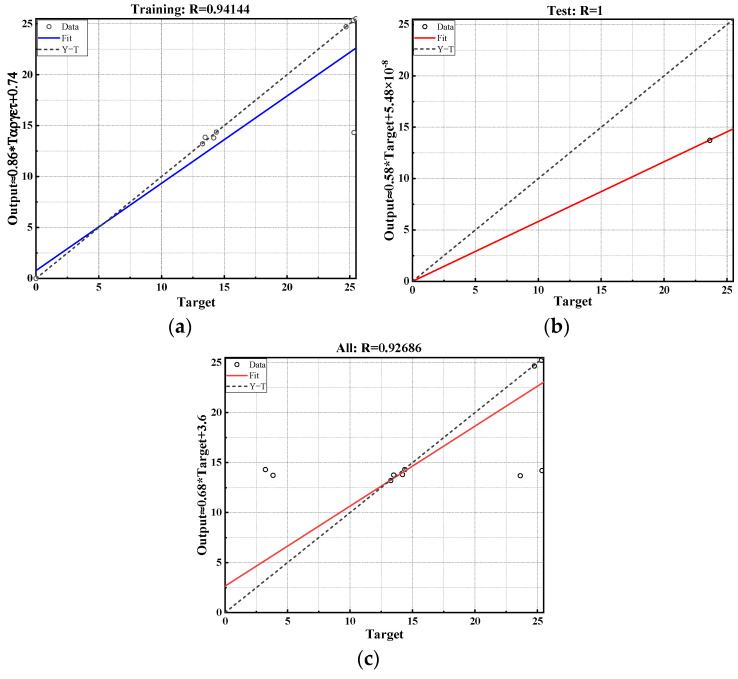
Prediction results of RBF neural network model: (**a**) training set; (**b**) test set; (**c**) overall set.

As shown in Figure 12, the regression coefficient of the RBF neural network model established in this study is 0.93216. This indicates that the prediction model has a high accuracy and can be used for predicting and analyzing flame retardancy. Based on this prediction model, the test results were calculated and analyzed, as shown in Figure 13.

As shown in Figure 13, the correlation coefficients for the training set, test set, and overall set are all greater than 0.9, indicating a strong correlation between the predicted values and the measured values of flame retardancy. This demonstrates that the RBF neural network model maintains good output stability alongside high prediction accuracy. The prediction model established in this study effectively predicts and evaluates flame retardancy, providing valuable references for the evaluation of WFM.

Additionally, although indicators such as combustion time, mass loss rate, and stability all indicate that the flame retardancy of the asphalt mixture is improved after modification, it is not possible to identify which evaluation indicator has the most significant impact on flame retardancy through test result analysis alone. Therefore, this study uses the PCA model to investigate the indicators [38,39]. An analysis model was established using IBM SPSS Statistics 25 software to perform a quantitative analysis of the influencing factors, and the combustion time, mass loss rate, and stability are selected as the variables (x_1_~x_3_). The results are shown in Table 6.

As shown in Table 6, the cumulative contribution rate of x_1_ and x_2_ is 96.289%, which is much larger than the requirement of 85%; thus, it is considered that x_1_ and x_2_ can explain the global variables, and x_1_ and x_2_ are selected to elaborate the model, which is shown in Table 7.

From Table 7, the score evaluation models of the two principal components are F_1_ = 0.515x_1_ + 0.51x_2_ − 0.069x_3_, F_2_ = 0.007x_1_ − 0.141x_2_ + 0.989x_3_, and the PCA model is calculated based on the contribution of each principal component model with its corresponding contribution rate, i.e., F = 0.629F_1_ + 0.334F_2_ = 0.327x_1_ + 0.274x_2_ + 0.374x_3_. Based on the weight coefficients, the most significant indicator affecting flame retardancy is combustion time. This is because it directly measures the duration of the asphalt mixture’s combustion under a fire source. A shorter combustion time indicates that the flame retardant in the asphalt mixture has effectively suppressed the combustion process, reducing the severity of the fire. Additionally, a shorter combustion time means that the combustion behavior has less impact on the service performance of the asphalt mixture, thereby significantly curbing the rate of loss of service performance. Therefore, in the study of the flame retardancy of warm mix flame retardant asphalt mixtures, special attention should be paid to the combustion time.

### 3.6. Analysis of Pavement Structural Dynamic Response

As noted in the previous analysis, the asphalt mixture prepared with the FR and EC exhibits good high-temperature performance and flame retardancy. However, its low-temperature performance and moisture stability are inferior to VAM. Although both meet the required performance standards, it is challenging to fully analyze their performance differences during service through conventional pavement performance tests. Therefore, this study uses ABAQUS 6.14-4 software to establish a three-dimensional finite element model of the pavement structure and analyze the dynamic response of pavements made from the two asphalt mixtures under vehicular load [40,41].

**Figure 14:**
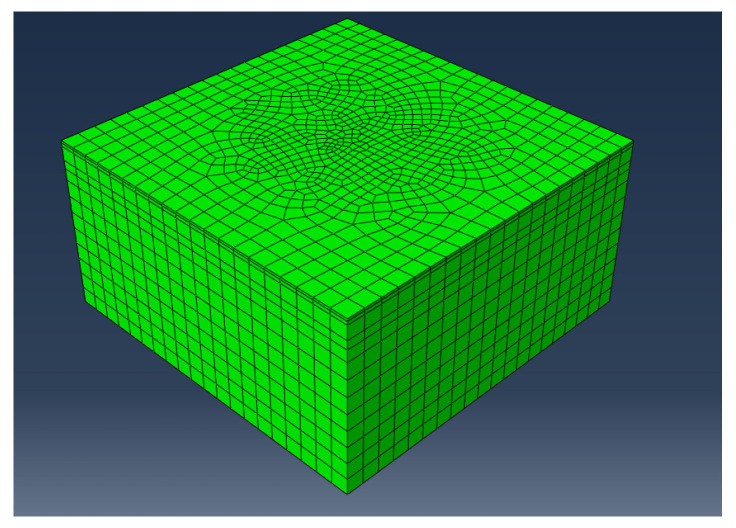
Finite element model after applying load and splitting the cells.

Considering the variety of vehicle types and the difficulty of standardizing axle loads, this study selects a dual-wheel single-axle load of 100 kN as the standard axle load. The load applied by the tires is 0.7 MPa. When a vehicle moves on the pavement, the tire’s shape is more akin to a circle. Referring to previous studies, it is assumed that the tire–pavement contact area is circular, with a diameter of 15 cm and a center-to-center distance of 50 cm. A three-dimensional finite element model with dimensions of 6 m × 6 m × 3 m is established using ABAQUS software, as shown in Figure 14.

**Figure 15:**
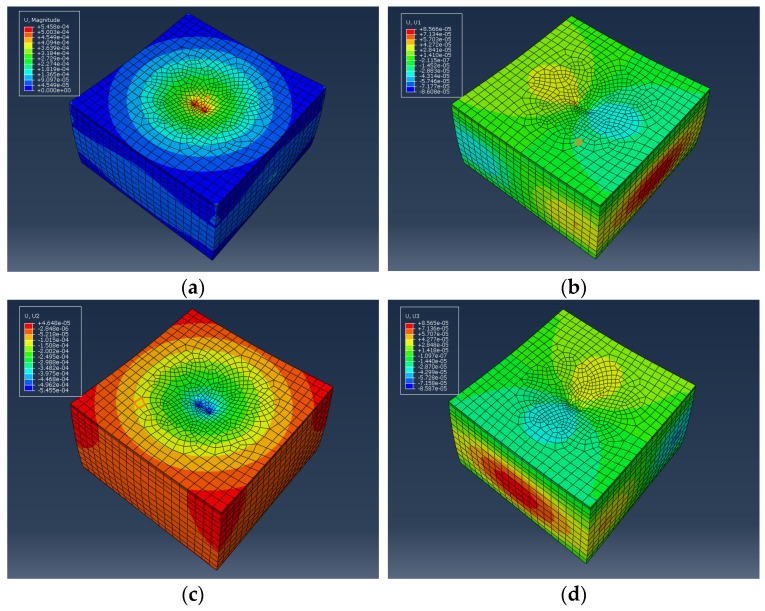
Displacement cloud diagram of VAM: (**a**) overall; (**b**) *X*-axis; (**c**) *Y*-axis; (**d**) *Z*-axis.

**Figure 16:**
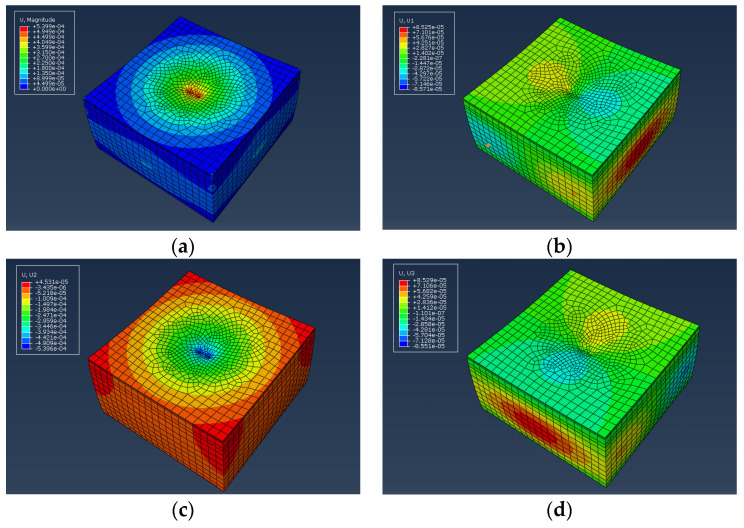
Displacement cloud diagram of WFM: (**a**) overall; (**b**) *X*-axis; (**c**) *Y*-axis; (**d**) *Z*-axis.

The established three-dimensional model is subjected to loads for calculations and the calculated data were analyzed, as shown in Figure 15 and Figure 16.

**Figure 17:**
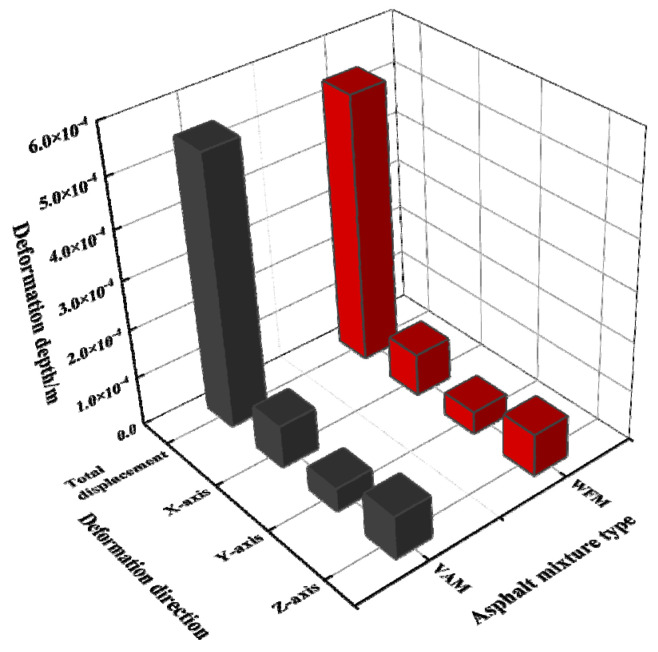
Deformation depth of asphalt mixture.

From Figure 15 and Figure 16, it can be seen that the maximum deformation depth of the pavement is located at the center of the two circular load areas. The maximum deformation depth for VAM is 5.458 × 10^−4^ m, while for WFM it is 5.399 × 10^−4^ m. Additionally, as one moves from the center to the outer edge of the circles, the deformation depth of the pavement decreases. Under the action of the dual circular load, the displacement cloud diagram gradually radiates outward from the center. A statistical analysis of the deformation depth in various directions is shown in Figure 17.

Comparing the deformation depth in different directions, it can be observed that the deformation depth of WFM is slightly lower than VAM. This indicates that although warm mix flame retardant modification may have some adverse effects on certain pavement performances of the mixture, the dynamic response analysis model under traffic load shows reduced deformation. This suggests that the designed warm mix flame retardant modification is beneficial for the service performance of the asphalt mixture.

## 4. Conclusions

This study investigates the design and performance of asphalt mixtures modified with a combination of FR and EC. After evaluating the pavement performance and flame retardancy of the asphalt mixtures, the flame retardancy of asphalt mixtures is predicted by an RBF neural network model and PCA model. Finally, a three-dimensional finite element model is established to analyze the effect of traffic load on the pavement structure, and the following conclusions are obtained:

(1) The optimal dosage of FR is 12%, and the optimal dosage of EC is 4%. The viscosity–temperature curves indicate that the mixing and compaction temperatures of the modified mixtures decreased by approximately 12 °C. This demonstrates that the addition of EC can reduce the mixing and compaction temperatures of asphalt mixtures, achieving the desired warm mix effect.
(2) After the warm mix and flame retardant modification of VAM, the high–temperature performance indicators of the mixture showed an increasing trend, while the low-temperature performance and moisture stability indicators exhibited a decreasing trend. This indicates that the high-temperature performance of the mixture is improved after the warm mix and flame retardant modification, but the low-temperature performance and moisture stability are attenuated.
(3) Considering the test results of combustion time, mass loss rate, and stability, the WFM exhibits a better flame retardancy compared to VAM, which indicates that the EC and FR used in this study significantly improve the flame retardancy of the asphalt mixtures.
(4) The RBF neural network model demonstrates that the flame retardancy prediction model established in this study has high accuracy, enabling effective evaluation of the flame retardancy of the asphalt mixtures. The PCA model reveals that combustion time has the most significant impact on flame retardancy. Therefore, it is important to carefully consider the effect of combustion time on the flame retardancy when designing a WFM.
(5) The finite element model reveals that the displacements in all directions of WFM are slightly smaller than VAM, indicating that the warm mix flame retardant modification method designed in this study benefits the service performance of the asphalt mixture.

## Figures and Tables

**Table 1:** Basic properties of VA.

Index	Test Value	Requirement	Test Method
Penetration at 25 °C/(0.1 mm)	55.6	40–60	T0604
Ductility at 5 °C/cm	363	≥20	T0605
Softening point/°C	74.1	≥60	T0606
Dynamic viscosity at 60 °C/(Pa/s)	2.52	—	T0620
Mass loss rate after RTFOT aging/%	0.48	−1.0–1.0	T0609

**Table 2:** Basic properties of aggregate.

Index	Test Value	Requirement	Test Method
Crushing value/%	9.6	≤26	T0316
Abrasion value/%	14.2	≤28	T0317
Apparent relative density/(g/cm^−3^)	2.817	≥2.60	T0304
Water absorption/%	0.75	<2	T0304

**Table 3:** Basic properties of EC.

Index	Test Value
Density/(g/cm^3^)	0.95
Melting point/°C	100
Flash point/°C	279
Solubility	Water immiscible
Appearance	Faint yellow granules

**Table 4:** Basic properties of several materials.

Material Type	Appearance	Density/(g/cm^3^)	Solubility	Toxicity
Magnesium hydroxide	White powder	2.38	Water immiscible	Non-toxic
Diatomite	White powder	2.26	Water immiscible	Non-toxic
Aluminum hydroxide	White powder	2.42	Water immiscible	Non-toxic

**Table 5:** Main components of VOC.

VA	Concentration/(ng/L)	Warm Mix Flame Retardant Asphalt	Concentration/(ng/L)
toluene	139.5	acrolein	87.3
acetone	62.1	acetone	65.9
p/m-xylene	53.4	propane	43.2.8
acrolein	53.2	n-butane	39.2
propane	48.5	ethane	35.9
propylene	41.6	n-pentane	32.3
ethane	40.7	propylene	24.3
n-butane	39.1	benzene	23.9
n-hexane	38.7	n-hexane	23.3
n-pentane	37.6	heptane	21.4

**Table 6:** Calculation results of PCA model.

Variable	Initial Eigenvalue			Extraction of the Square Sum of Loads		
	Total	Variance Percentage/%	Cumulative/%	Total	Variance Percentage/%	Cumulative/%
x_1_	1.887	62.893	62.893	1.887	62.893	62.893
x_2_	1.002	33.396	96.289	1.002	33.396	96.289
x_3_	0.111	3.711	100			

**Table 7:** Initial factor load matrix.

Variable	Principal Component 1	Principal Component 2	Score Coefficient 1	Score Coefficient 2	Weight Coefficient
x_1_	0.972	0.007	0.515	0.007	0.363438612
x_2_	0.962	−0.141	0.51	−0.141	0.319604899
x_3_	0.13	0.991	0.069	0.989	0.316956489

## Data Availability

The original contributions presented in the study are included in the article, further inquiries can be directed to the corresponding author.
